# Inherently Chiral Calixarenes: Synthesis, Optical Resolution, Chiral Recognition and Asymmetric Catalysis

**DOI:** 10.3390/ijms12010429

**Published:** 2011-01-17

**Authors:** Shao-Yong Li, Yao-Wei Xu, Jun-Min Liu, Cheng-Yong Su

**Affiliations:** 1 School of Chemistry and Chemical Engineering, Sun Yat-Sen University, Guangzhou 510275, China; E-Mails: mylatulipe@yahoo.com.cn (Y.-W.X.); cesscy@mail.sysu.edu.cn (C.-Y.S.); 2 College of Pharmacy, Tianjin Medical University, Tianjin 300070, China; E-Mail: lishaoyong@tijmu.edu.cn

**Keywords:** inherently chiral calixarene, synthesis, optical resolution, chiral recognition, asymmetric catalysis

## Abstract

Inherently chiral calixarenes, whose chirality is based on the absence of a planar symmetry or an inversion center in the molecules as a whole through the asymmetric array of several achiral groups upon the three-dimensional calix-skeletons, are challenging and attractive chiral molecules, because of their potential in supramolecular chemistry. The synthesis and optical resolution of all varieties of inherently chiral calixarenes are systematically discussed and classified, and their applications in chiral recognition and asymmetric catalysis are thoroughly illustrated in this review.

## 1. Introduction

As the third generation of supramolecules, after cyclodextrins and crown ethers, calixarenes have been extensively studied for host-guest chemistry and frequently used in separations, as sensors and catalysts, because of their unique cavity-shaped architecture and preorganized binding sites [1]. The addition of chirality to calixarenes is an exciting enhancement of their already robust potential as ligands for chiral catalysis and enantioselective separations. Chiral calixarenes can be easily produced by the attachment of a chiral moiety on calixarene skeletons by virtue of the fact that no resolution procedure is required. Therefore, many chiral calixarenes containing chiral residues at either the lower or upper rim have been prepared as chiral receptors [2–8] and catalysts [9–11]. However, introducing “inherent chirality” into calixarenes by asymmetric placement of achiral substituents on their skeletons is more challenging and attractive.

**Figure 1:**
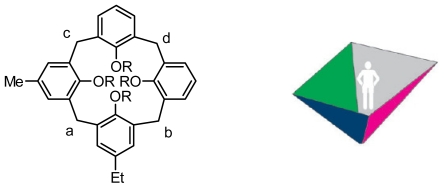
Proposal of a chirality descriptor for inherent chirality.

The concept of “inherently chiral” was first suggested by Böhmer [12] and formulated by Schiaffino [13]. Inherent chirality was viewed as “arising from the introduction of a curvature in an ideal planar structure that is devoid of symmetry axes in its bidimensional representation” (axes positioned in the two-dimensional plane, as explained by Schiaffino). Subsequently, Szumna made a minor modification in which “inherent chirality arises from the introduction of a curvature in an ideal planar structure that is devoid of perpendicular symmetry planes in its bidimensional representation” [14]. Moreover, two type of notations, (*cR*)/(*cS*) and (*P*)/(*M*), were recommended to describe inherent chirality by Schiaffino and Szumna, respectively. In the case of calix[4]arenes, once the carbons of the bridges are labeled as a, b, c, and d according to standard stereochemistry rules, an ideal observer standing on the concave side of the surface will see the three highest priority atoms a, b, and c. The (*cR*)/(*cS*) chirality description designates a clockwise priority of their sequence as *cR* and counterclockwise priority of their sequence as *cS*, respectively, in which *c* stands for curvature. Correspondingly, the (*P*)/(*M*) one does it as *P* or *M*, respectively (Figure 1). The latter notation was highly recommended for inherently chiral calixarenes by Szumna because they are typical in concave molecules, including salphen complexes, cyclic amides, derivatives of sumanene, trioxatricornan or subphthalocyanine, cyclotriveratrylenes, homooxacalix[3]arenes, resorcinarenes, phthalocyanines, corannulenes, and cavitands.

Since the first example of inherently chiral calyx[4]arene appeared in 1982 [15], numerous studies have reported the synthesis of various inherently chiral calixarenes and their applications in chiral recognition and asymmetric catalysis. Some special reviews on inherently chiral calixarenes are currently available in the literature. Böhmer first described various attempts to transform calixarenes into molecules with inherent chirality [12]. Huang and Chen then reviewed the synthesis and optical resolution of inherently chiral calixarenes and their applications in enantioselective recognition and asymmetric catalysis [16]. Recently, inherently chiral calixarenes were simply reviewed by Szumna with regard to their synthesis and applications as a representative inherently chiral concave molecule [14]. Moreover, McIldowie described examples of *Cn*-dissymmetric calixarenes based on their stereochemical significance and discussed aspects of their chirality [17]. In the past decade, the research area dealing with inherently chiral calixarenes rapidly expanded, and many novel examples of their synthesis and applications in chiral recognition and asymmetric catalysis subsequently appeared. The present review attempts to provide a systematic overview of inherently chiral calixarenes and their recent developments.

## 2. Synthesis of Inherently Chiral Calixarenes

Fragment condensation with different phenolic units and asymmetric functionalization of the calixarene skeleton are two primary ways to synthesize inherently chiral calixarenes. The former is not popular because of its tedious steps and lower yields, although it is principally versatile and only used to prepare a few inherently chiral calyx[4]arenes in the early stage. No extant report has presented fragment condensation in the synthesis of larger inherently chiral calixarenes.

Conversely, asymmetric functionalization of the calixarene skeleton is widely used to construct inherently chiral calixarenes. Chiral induction elements based on this method include conformation and functionalization position. Four typical conformations (cone, partial cone, 1,2-alternate, and 1,3-alternate) are adopted by calixarene, especially calix[4]arene. *O*-Alkylation or esterification of phenolic OH on the lower rim, and *para*- and *meta*-substitution of phenolic units on the upper rim, are often used to asymmetrically functionalize calixarene when its macrocycle is unopened. Conformational variation combined with functionalization at different positions produces a variety of inherently chiral calixarenes. Until now, most inherently chiral calix[4]arenes and all of inherently chiral calix[5]arenes, calix[6]arenes, and calix[8]arenes are prepared in this method.

### 2.1. Fragment Condensation

**Figure 2:**
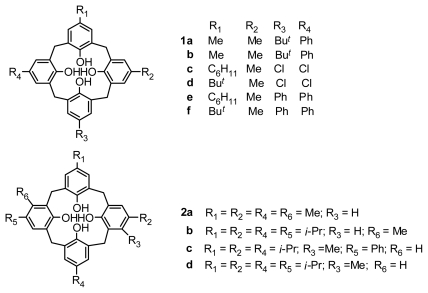
Inherently chiral calix[4]arenes from fragment condensation.

Inherently chiral calix[4]arenes can be synthesized through [3 + 1] or [2 + 2] fragment condensation with different *para*-substituted phenolic units. The typical example of inherently chiral calix[4]arenes **1a–f** (Figure 2), consisting of three different phenolic units, was prepared by fragment condensation either of a trinuclear compound with a 2,6-bis(bromomethyl)phenol or a dinuclear compound with a bisbromomethylated dimer by Böhmer [18]. Inherently chiral calix[4]arenes can also be synthesized through fragment condensation with different *meta*-substituted phenolic units. Royer synthesized these type of inherently chiral calix[4]arenes **2a–d** (Figure 2) with linear phenolic trimers and 2,6-bis-bromomethylphenols with different *meta*-substituents [19]. Additionally, the one-pot synthesis method from one *meta*-substituted monomer can result in inherently chiral calix[4]arenes with *C_2_* or *C_4_* symmetry, which were exemplified by those entities reported by Böhmer [20].

### 2.2. Asymmetric Functionalization

#### 2.2.1. Asymmetric Functionalization on the Lower Rim

**Figure 3:**
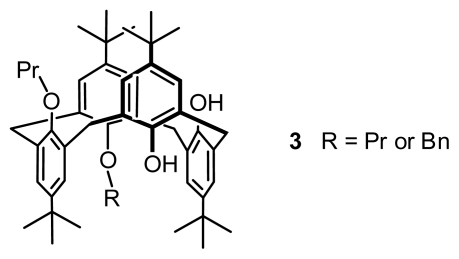
*O*-Substituted inherently chiral calix[4]arene in 1,3-alternate conformation.

**Figure 4:**
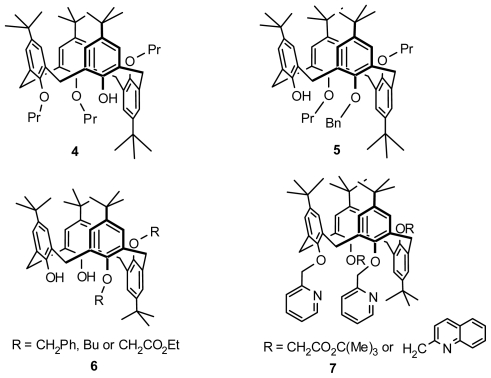
*O*-Substituted inherently chiral calix[4]arenes in partial cone conformation.

All possible chiral isomers that can be derived from calix[4]arene by modification of the OH groups were systematically classified by Shinkai [21]. The numbers of chiral isomers are 24 for tetra-*O*substituted calix[4]arenes, 10 for tri-*O*-substituted calix[4]arenes, 3 for di-*O*-substituted calix[4]arenes, and 0 for mono-*O*-substituted calix[4]arenes. To demonstrate asymmetry in these chiral calix[4]arenes, they synthesized several tetra-, tri-, and di-*O*-substituted calix[4]arenes, in which **3** (Figure 3) comprises the first di-*O*-substituted inherently chiral calix[4]arene in 1,3-alternate conformation, and **4** and **5** (Figure 4) are the first two tri-*O*-substituted entities in partial cone conformation.

In addition to the tri-*O*-substituted inherently chiral calix[4]arene in partial cone conformation above, Narumi and Miyano first obtained a di-*O*-substituted inherently chiral calix[4]arene in partial cone conformation. Proximally *O*,*O*′-disiloxane-bridged calix[4]arene was treated with benzyl bromide and desilylated to yield *anti*-*O*,*O*′-dibenzylcalix[4]arene **6** (Figure 4) [22]. A tetra-*O*-alkylated inherently chiral calix[4]arene in partial cone conformation **7** (Figure 4) reported by Pappalardo was synthesized from exhaustive alkylation of *syn*-proximal bis[(2-pyridylmethyl)oxy]calix[4]arene with *t*-butyl bromoacetate or 2-(chloromethyl)quinoline hydrochloride [23].

**Figure 5:**
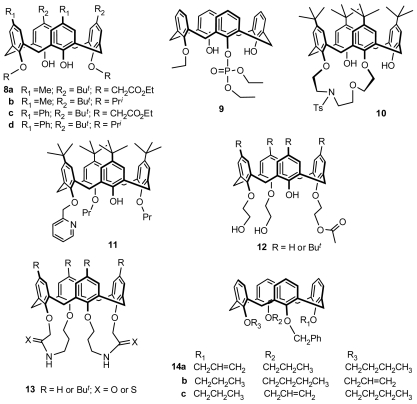
*O*-Substituted inherently chiral calix[4]arenes in cone conformation.

Di-*O*-substituted inherently chiral calix[4]arenes **8a–d** (Figure 5) in cone conformation were easily prepared by Böhmer from the alkylation of two distal phenolic hydroxyl groups of calix[4]arenes with two different phenolic units, whose chirality is derived from asymmetric superposition of substituents on the lower rim and upper rim [24]. Another type of di-*O*-substituted inherently chiral calix[4]arene in cone conformation was prepared by Kalchenko. *O*,*O*′-Phosphorotropic migration of the phosphoryl group from the distal to the proximal position of calix[4]arene produced an inherently chiral calix[4]arene phosphate **9** (Figure 5) in cone conformation [25]. Recently, we developed a one-step procedure to synthesize inherently chiral *p*-*tert*-butylcalix[4]azacrown **10** (Figure 5) in cone conformation through “1 + 1” crown etherification from *p*-*tert*-butylcalix[4]arene and tri-tosylated 2-(2-(2-hydroxyethoxy)ethylamino)ethanol, in which the two tosylate groups are not equivalent because of the existence of an *N*-tosyl group [26].

Inherently chiral calix[4]arene from tri-*O*-alkylation on the lower rim in cone conformation was first reported by Shinkai. *p*-*tert*-Butylcalix[4]arene was monoetherificated with 2-chloromethylpyridine hydrochloride, and subsequently dietherificated with propyl bromide, to yield an inherently chiral calix[4]arene **11** (Figure 5) [27]. Additionally, asymmetric tri-*O*-substitution on the lower rim can be achieved using enzymatic catalysis. McKervey used lipase-catalyzed transesterification to produce desymmetrization, producing inherently chiral calix[4]arene **12** (Figure 5) in cone conformation, with enantiomer excesses of up to 100% [28].

One type of tetra-*O*-alkylated inherently chiral calix[4]arene **13** (Figure 5) in cone conformation with double carboxamide bridges spanning the proximal positions on the lower rim was synthesized by double intramolecular cyclization when the relationship between ring closure and chain length of open chain precursors was studied by Bitter [29]. Subsequently, another type of tetra-*O*-alkylated inherently chiral calix[4]arenes **14a–c** (Figure 5) in cone conformation with four different substituents was synthesized by Chung and Lin using a general synthetic strategy that comprised a six-step sequence, in which monoalkoxycalix[4]arenes were treated with different alkyl halides at various alkylation stages [30].

**Figure 6:**
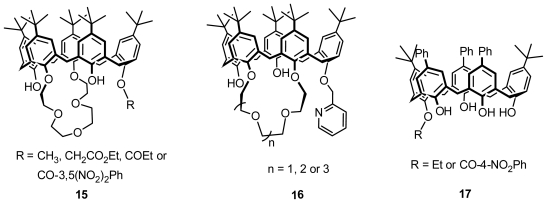
*O*-Substituted inherently chiral calix[5]arenes.

Until now, the reported inherently chiral calix[5]arenes are all prepared from asymmetric functionalization of calix[5]arene on the lower rim. Böhmer and Pappalardo produced an inherently chiral calix[5]arene 1,3-crown ether **15** (Figure 6) by *O*-alkylation or *O*-acylation of one of the two adjacent hydroxy groups of the *p*-*tert*-butylcalix[5]arene derivative, which was 1,3-di-*O*-etherificated with suitable oligoethylene glycol ditosylates. The strategy of introducing *O*-alkyl residues can be inverted. Monoalkylation of *p*-*tert*-butylcalix[5]arene with 2-(chloromethyl)pyridine after reacted with suitable oligoethylene glycol ditosylates afforded inherently chiral calix[5]arene 1,2-crown ether **16** (Figure 6) [31]. No also synthesized inherently chiral calix[5]arene **17** (Figure 6) by mono-*O*-ethylation or benzoylation of calix[5]arene prepared from [3 + 2] fragmentation condensation between bishydroxymethylated dimer of *p*-phenylphenol and *p*-substituted phenol trimer [32].

**Figure 7:**
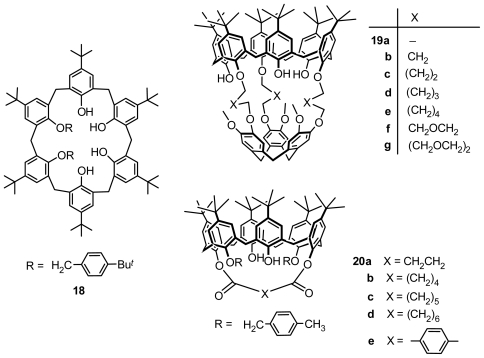
*O*-Substituted inherently chiral calix[6]arenes.

Asymmetrical *O*-substitution on the lower rim is also applied to prepare inherently chiral calix[6]arene. Inherently chiral *anti* isomers of l,2-bis-(*p*-*tert*-butylbenzyl) ether of *p*-*tert*butylcalix[ 6]arene **18** (Figure 7) was obtained by Neri by heating a tetrachloroethylene solution of its *syn* isomer, which is a mixture of various conformers of the same class in a fast exchange regime at room temperature [33]. A type of inherently chiral cryptocalix[6]arenes **19a–g** (Figure 7) with *C_3_* symmetry was first synthesized by Reinhoudt by covalent three-point linking of *p*-*tert*-butylcalix[6]arene to a cyclotriveratrylene [34]. Moreover, Gutsche designed 1,4-ditolyl ether of diester-bridged *p*-*tert*-butylcalix[6]arenes **20a–e** (Figure 7) synthesized from *p*-*tert*-butylcalix[6]arene 1,4-ditolyl ether and appropriate diacid chloride, which were inherently chiral with *C_2_* symmetry [35].

**Figure 8:**
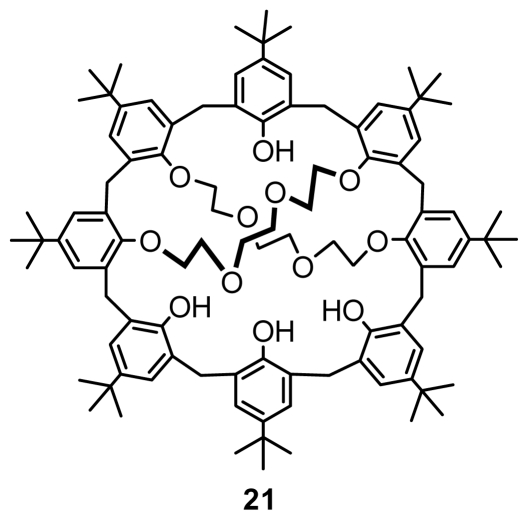
*O*-Substituted inherently chiral calix[8]arene.

Similar to inherently chiral calix[5]arene, all inherently chiral calix[8]arenes were also reportedly derived from asymmetric functionalization of calix[8]arene on the lower rim. A typical example of covalently linked, inherently chiral calix[8]arene 1,4-2,5-calix[8]biscrown-4 **21** (Figure 8) was obtained by Neri from either 1,4-calix[8]crown-4 or *p*-*tert*-butylcalix[8]arene by alkylation with triethylene glycol ditosylate [36].

#### 2.2.2. Asymmetric Functionalization on the Lower and Upper Rims

**Figure 9:**
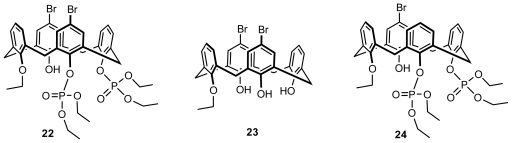
Inherently chiral calix[4]arenes in cone conformation asymmetrically functionalized on the lower and upper rims.

Based on *O*,*O*′-phosphorotropic rearrangements of 1,3-distally disubstituted calix[4]arenes into 1,2-proximal isomers, regioselective bromination of the upper rim, and hydrolytic removal of the phosphoryl or benzoyl group from the lower rim, Kalchenko first synthesized three types of inherently chiral calix[4]arenes **22–24** (Figure 9) in cone conformation with asymmetrical superposition of ethyl, diethoxyphosphoryl groups on the lower rim, and bromine atoms on the upper rim [25].

**Figure 10:**
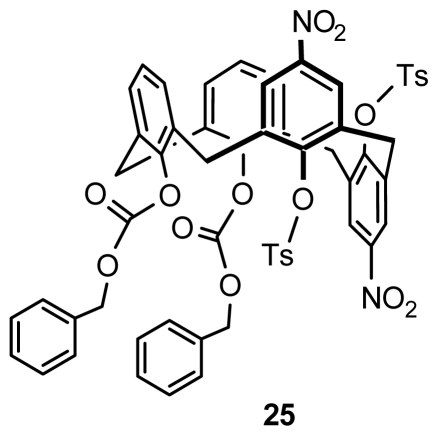
Inherently chiral calix[4]arene in partial cone conformation asymmetrically functionalized on the lower and upper rims.

Hesek found that benzyloxycarbonyl substitution of calix[4]arene on the lower rim, intramolecularly rearranged from the 1,3-position to 1,2-positon when nitrated with HCl, NaNO_3_, and a catalytic amount of Ac_2_O, produced a new type of inherently chiral calix[4]arene **25** (Figure 10) in partial cone conformation with asymmetric functionalization on the lower rim and the upper rim after tosylation [37]. But further optimization of the reaction conditions to maximize its yield was not performed.

**Figure 11:**
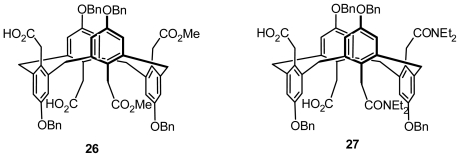
Inherently chiral calix[4]arenes in 1,3-alternate conformation asymmetrically functionalized on the lower and upper rims.

Inherently chiral calix[4]arenes in 1,3-alternate conformation with asymmetric functionalization on the lower and upper rims were presented by Gutsche *et al.* [38]. The authors alcoholyzed or aminated the bisanhydrides from tetrabenzyl ether, tetra-*p*-bromobenzenesulfonate, and tetra-*p*-methylbenzenesulfonate of *p*-carboxymethylcalix[4]arene in the 1,3-alternate conformation to yield inherently chiral calix[4]arenes carrying two carboxymethyl and two carboalkoxymethyl groups on the upper rim, or inherently chiral calix[4]arenes carrying two carboxymethyl and two amidomethyl groups on the upper rim. **26** and **27** are illustrated in Figure 11 as typical examples of these two types of inherently chiral calix[4]arenes.

#### 2.2.3. Asymmetric Functionalization on the Upper Rim

**Figure 12:**
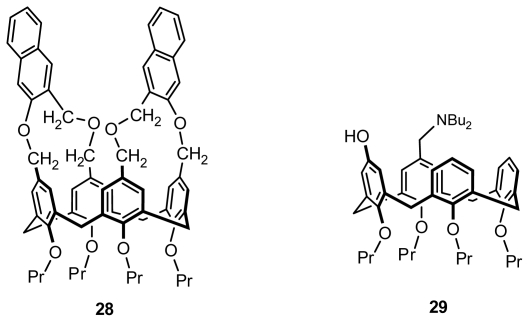
Inherently chiral calix[4]arenes in cone conformation asymmetrically functionalized on the upper rim.

The earliest inherently chiral calix[4]arene was accidentally prepared through asymmetric functionalization on the upper rim by Gutsche *et al.* in 1982, which was a prelude to the study of inherently chiral calixarenes, although its conformation was not identified [15]. After a few years, Shinkai reported that a cross-link reaction between *p*-chloromethylcalix[4]arene tetrapropyl ether and 3-hydroxymethyl-2-naphthol in the presence of base resulted in a *syn* isomer and *anti* isomer **28** (Figure 12), the latter one classified as an inherently chiral calix[4]arene [39]. Another typical inherently chiral calix[4]arene **29** (Figure 12) in cone conformation asymmetrically functionalized on the upper rim was synthesized from proximally *p*-dibrominated tetra-*O*-propylcalix[4]arene by Shimizu [40]. Subsequently, they prepared a series of similar entities and studied their properties.

**Figure 13:**
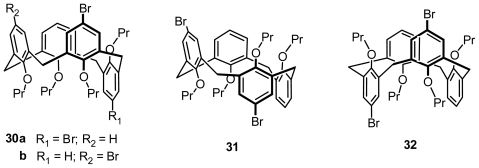
Inherently chiral calix[4]arenes in noncone conformation asymmetrically functionalized on the upper rim.

All six possible conformational isomers of the proximally *p*-dibrominated calix[4]arene tetraalkyl ether were selectively synthesized by appropriate control of stereochemistry during di-*O-*alkylation reactions of 5,11-dibromocalix[4]arene *syn*-dialkyl ethers by Shimizu, in which four conformational isomers of 5,11-dibromocalix[4]arene tetrapropyl ether, **30a**, **30b**, **31**, and **32** (Figure 13), namely two partial, one 1,2-alternate, and one 1,3-alternate conformers, are inherently chiral [41].

**Figure 14:**
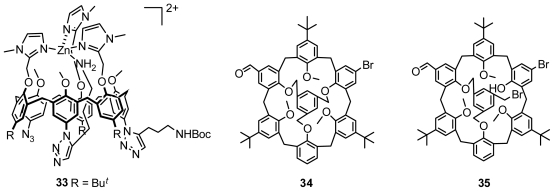
Inherently chiral calix[6]arenes in cone conformation asymmetrically functionalized on the upper rim.

Asymmetric functionalization of calix[6]arene on the upper rim is another method to produce inherently chiral calix[6]arene. Reinaud demonstrated an interesting and novel inherently chiral calix[6]arene **33** (Figure 14) in cone conformation, which was prepared with an efficient method for the controlled, stepwise functionalization of calix[6]arene on the upper rim associated with metal-template and molecular inclusion [42]. Moreover, we recently developed an efficient route to synthesize inherently chiral calix[6]arene **34** and its intermediate **35** (Figure 14) in cone conformation from selective formylation and bromination of 1,3,5-bridged calix[6]arene with a mesitylenyl group on the upper rim [43].

#### 2.2.4. Asymmetric Functionalization on Phenolic *Meta*-Position

**Figure 15:**
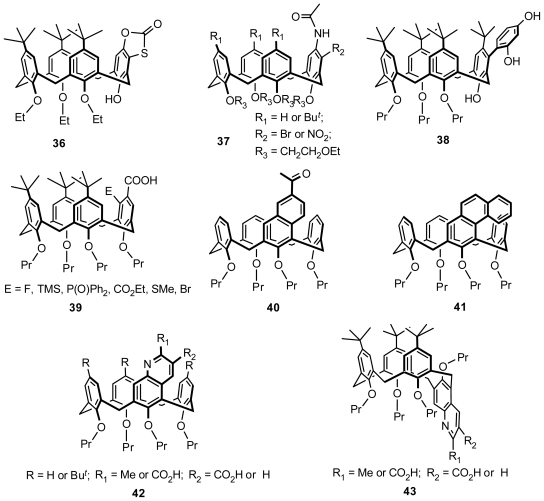
Inherently chiral calix[4]arenes asymmetrically functionalized on the phenolic *meta*-position.

Gutsche synthesized inherently chiral calix[4]arenes with a *meta-*substituted phenolic unit, such as **36** (Figure 15) in cone conformation as a typical example, from 1,4-conjugate additions of calix[4]arene monoquinone with a variety of nucleophiles, including sodio diethyl malonate, acetate, thiourea, *p*-thiocresol, and mercaptoacetic acid, when they studied the synthesis possibilities of calix[4]quinone, which provided a facile route to synthesize inherently chiral calix[4]arenes [44]. A simple and high-yield approach for preparing *meta*-substituted inherently chiral calix[4]arenes from *para*-acetamido substituted calix[4]arenes was reported by Reinhoudt. The experiments demonstrated that electrophilic substitution at the *meta*-position is favored over the free *para*-positions of the other aromatic rings because of the activation of *para*-acetamido under carefully controlled conditions [45]. The inherently chiral calix[4]arene **37** prepared using this approach from mono(*para-*acetamido) calix[4]arenes is shown in Figure 15. The introduction of aromatic moieties at the calixarene upper rim represents a novel procedure to obtain calix[4]arenes with an enlarged aromatic central cavity. A series of inherently chiral calix[4]arenes, with a typical example **38** (Figure 15), asymmetrically aromatizated on the phenolic *meta*-position, were prepared by Gaeta and Neri through dienone-phenol rearrangement on calixarene [46].

A chiral auxiliary can adhere to the *para*-position of calixarene and envisaged to induce an asymmetrical substitution when incorporating a substituent at its *meta*-position. General asymmetric synthesis of inherently chiral calix[4]arenes was first described by Arnott [47]. Using a chiral oxazoline derived from l-valine, an ortholithiation strategy was employed to yield inherently chiral calix[4]arene **39**·(Figure 15) with a high (93%) enantiomeric excess. Although Huang and Chen also introduced a chiral auxiliary, l-Boc-prolinyl amide, on the phenolic *para*-position before *meta*-nitration, the separated diastereomers exhibited almost no asymmetric induction [48].

In addition to incorporating a substituent on the *meta*-position, another approach based on the transformation of calixarene’s phenyl into naphthalenyl, quinolinyl, or phenanthrenyl by intramolecular ring closure is also applied to create inherently chiral calixarenes. These typical examples were produced by Shinkai [39], Dyker [49], and Huang and Chen [50], which are shown as **40**, **41**, **42**, and **43** (Figure 15).

## 3. Optical Resolution of Inherently Chiral Calixarenes

Since Gutsche reported the first inherently chiral calixarene almost three decades ago, there has been an increasing interest in their study for their unique chiral structures and potential applications in chiral recognition and asymmetric catalysis. In the preliminary stage, most attempts focused on their synthesis and optical resolution because their optical resolution was usually achieved through high-performance liquid chromatography (HPLC) methods, which was inappropriate for scale-up, and thus impeded their practical applications. However, in recent years, many types of optical resolution methods have been successfully developed to resolve inherently chiral calixarenes on the gram scale, which substantially promoted their application in chiral recognition and asymmetric synthesis. Their optical resolution is separately enumerated herein because they are so crucial for the progress of the entire field. Similar to other types of chiral molecules, inherently chiral calixarenes can be optically resolved with the following conventional chiral resolution methods.

### 3.1. Resolution by Chiral Column Chromatography

**Figure 16:**
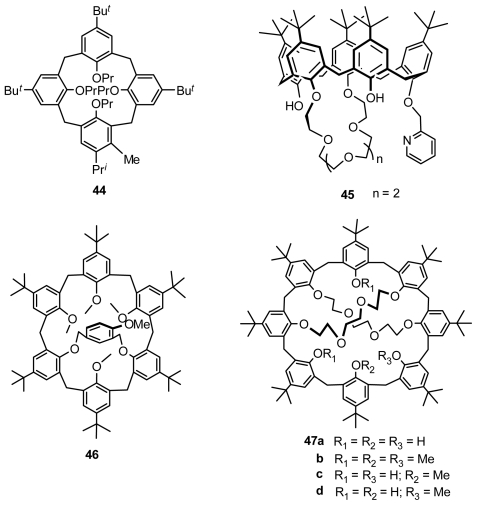
Inherently chiral calixarenes resolved with chiral column chromatography.

HPLC or LC with a chiral column was widely applied in preliminary attempts at theoptical resolution of many types of inherently chiral calixarenes. Shinkai first resolved racemic inherently chiral calix[4]arene **44** (Figure 16) from fragment condensation using an LC method with a chiral packing column (Daicel Chiralpak OP[+]) [51] and racemic inherently chiral calix[4]arene **11** from asymmetric functionalization using an HPLC method with a chiral packing column (Sumipax OA-2000) [27].

Compared with inherently chiral calix[4]arenes, larger inherently chiral calixarenes are rarely optically resolved by HPLC with a chiral column. Pappalardo successfully resolved inherently chiral calix[5]arene 1,2-crown ether **15** and inherently chiral calix[5]arene 1,3-crown ether **45** (Figure 16) by HPLC with a Chiralpak AD column and Chiralcel OD column [52]. To find definitive evidence for the immobilization of the calix[6]arene ring, Shinkai synthesized inherently chiral calix[6]arene **46** (Figure 16), the 1,3-phenyl units of which are bridged by an asymmetric 4-methoxy-*m*-xylenyl unit, and optically resolved it by an HPLC method with a chiral packed column [53]. Neri and Caccamese resolved 1,4-2,5-calix[8]bis-crown-4 and its methyl derivative **47** (Figure 16) by HPLC with a Chiralpak AD column and Chiralcel OD column [54].

### 3.2. Resolution from Diastereomers by Conventional Chromatography

**Figure 17:**
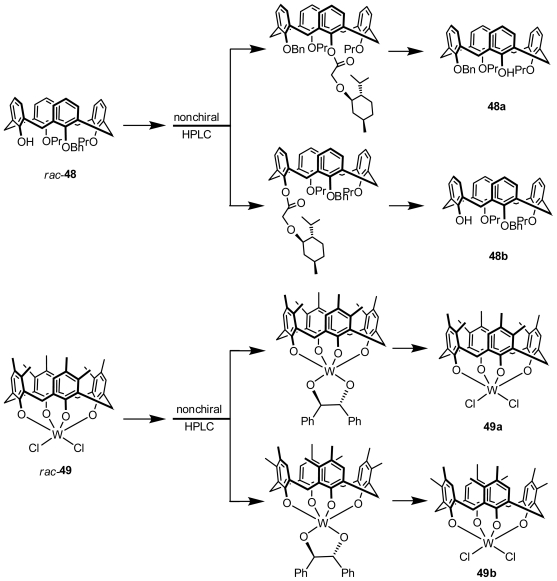
Inherently chiral calix[4]arenes resolved from diastereomers by conventional column chromatography.

Inherently chiral calixarenes can be indirectly resolved through separation of the diastereomers formed with a chiral auxiliary and their racemates by conventional chromatography (flash column chromatography, preparative TLC or HPLC with a nonchiral column) and removal of the chiral auxiliary. The first example of a preparation of enantiopure inherently chiral calix[4]arenes was reported by Shinkai. Inherently chiral calix[4]arene **48** (Figure 17) was esterificated with (−)-menthoxyacetyl chloride to yield a pair of corresponding diastereomers, which could be separated by nonchiral HPLC. A pair of enantiomeric antipodes, **48a** and **48b**, was successfully obtained after hydrolysis of the separated diastereomers [21]. Subsequently, Huang and Chen achieved a series of resolutions of inherently chiral calix[4]crown derivatives with chiral auxiliary *S*-BINOL and found that both the size of the crown moiety and alkylation of the last phenolic hydroxy group (with or without a change in conformation) affect the separation of the diastereomers [55,56].

Swager described an example of optical resolution of an inherently chiral metallocalix[4]arene. Dichlorotungsten (VI) calix[4]arene complex **49** (Figure 17), prepared from 3,4-dimethylcalix[4]arene and WCl_6_, was coupled with (1*S*,2*S*)-1,2-diphenylethane-1,2-diol to prepare a (*S*,*S*)-(−)-hydrobenzoinbased disastereomeric mixture, which was separated by nonchiral HPLC. The enantiomeric dichlorotungsten (VI) calix[4]arene complexes **49a** and **49b** were recovered from the separated diastereomers with AlCl_3_ [57].

**Figure 18:**
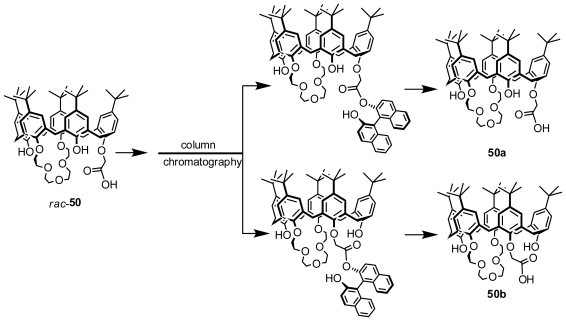
Inherently chiral calix[5]arenes resolved from diastereomers by conventional column chromatography.

Flash column chromatography was also successfully applied to resolve inherently chiral calix[5]arenes. Huang and Chen obtained a pair of enantiopure inherently chiral calix[5]arenes, **50a** and **50b** (Figure 18), which were prepared by separation of their diastereomers formed with *R*-BINOL and their racemates by column chromatography instead of HPLC and hydrolysis of the diastereomers [58].

### 3.3. Resolution through Recrystallization with a Chiral Auxiliary

**Figure 19:**
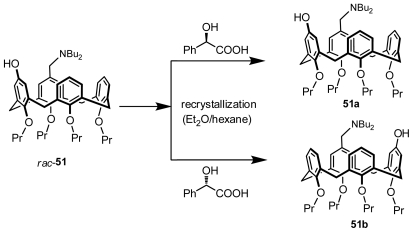
Inherently chiral calix[4]arenes resolved through recrystallization with a chiral auxiliary.

Inherently chiral calixarenes could be directly optically resolved from recrystallization of diastereomeric complexation without covalent bonding with chiral auxiliaries. Until now, only one example of an inherently chiral calix[4]arene has been optically resolved with this method, although it is popular in the optical resolution of other chiral molecules. Shimizu first prepared a pair of enantiomeric inherently chiral calix[4]arenes, **51a** and **51b** (Figure 19), by recrystallization after complexation between their racemate **51** and chiral mandelic acid in the Et_2_O and hexane mixture [40].

### 3.4. Resolution through Recrystallization of Diastereomers

**Figure 20:**
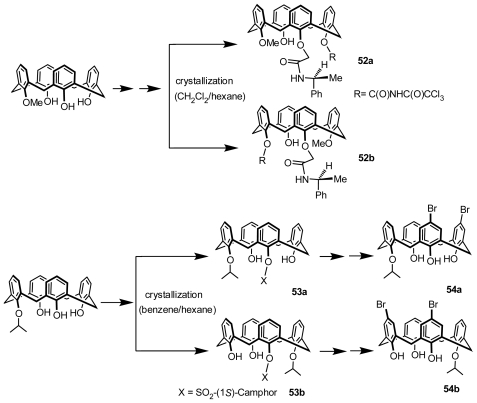
Inherently chiral calix[4]arenes resolved through recrystallization of diastereomers.

As an effective and economical method for replacing separation of diastereomers with conventional chromatography, recrystallization of diastereomers can also be applied to resolve inherently chiral calixarenes. This method was successfully applied to two separate examples of inherently chiral calix[4]arene diastereomers by Kalchenko. A pair of inherently chiral calix[4]arene diastereomers, **52a** and **52b** (Figure 20), synthesized from mono-*O*-methylcalix[4]arene, were obtained in analytical purity by crystallization from a mixture of CH_2_Cl_2_ and hexane. Alkylations of their OH groups, followed by removal of the amido and carbamate groups, generated a virtually unlimited structural and functional diversity of inherently chiral calix[4]arenes asymmetrically functionalized on the lower rim [59]. Subsequently, Kalchenko synthesized another pair of diastereomers, **53a** and **53b** (Figure 20), from mono-*O*-isopropylcalix[4]arene and successfully resolved them by simple crystallization from a mixture of benzene and hexane. The separated diastereomers were then brominated and hydrolyzed to produce targeted chiral molecules **54a** and **54b** (Figure 20) [60].

### 3.5. Kinetic Resolution

**Figure 21:**
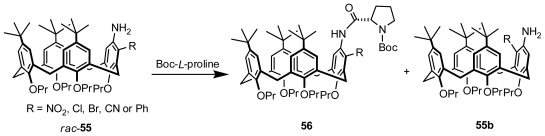
Kinetic resolution of inherently chiral calix[4]arenes.

An example of an effective kinetic resolution of racemic inherently chiral calix[4]arene was reported by Huang and Chen (Figure 21). Racemic *m*-nitro-substituted inherently chiral aminocalix[4]arene **55** (R = NO_2_) was transformed into a mixture of the corresponding amide **56** (R = NO_2_) and the remaining aminocalix[4]arene **55b** (R = NO_2_) when acylated with Boc-l-proline, which revealed the occurrence of nonenzymatic kinetic resolution. The influences of the ratio of acylating reagent to substrate, reaction time, and solvent on the resolution process were exhaustively studied [61].

To examine the universality of this kinetic resolution, several analogs (R = Cl, Br, CN, Ph, or NMe_2_) of **55** (R = NO_2_) were subsequently synthesized and resolved through acylation with Boc-l-proline. As expected, similar resolution results occurred to most of its analogs (R = Cl, Br, CN, or Ph), with the exception of one (R = NMe_2_) (Figure 21). The electron-withdrawing ability of the substituent was helpful for improving the resolution efficiency of the acylation process. Moreover, Cbz-l-proline and Boc-d-proline were shown to be efficient as acylation reagents to resolve racemic *m*-nitro-substituted inherently chiral aminocalix[4]arene **55** (R = NO_2_) [62].

## 4. Applications of Inherently Chiral Calixarenes

Chiral phenomena, including chiral recognition and asymmetric catalysis, are universal in biochemical systems. The study of chiral recognition and asymmetric catalysis based on artificial chiral receptors could contribute to their understanding. As more challenging and attractive chiral molecules, inherently chiral calixarenes obviously have great potential within this scope. However, only a few reports have presented the applications of inherently chiral calixarenes because most efforts have focused on their synthesis and enantiomeric resolution. Fortunately, their applications in chiral recognition and asymmetric catalysis successively appeared in recent years along with gram-scale resolution of enantiomeric inherently chiral calix[4]arenes. Until now, all of the applications in chiral recognition and asymmetric catalysis thoroughly arise from inherently chiral calix[4]arenes, although larger inherently chiral ones were also successfully resolved optically.

### 4.1. Chiral Recognition of Inherently Chiral Calixarenes

**Figure 22:**
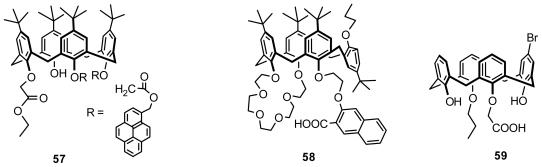
Inherently chiral calix[4]arenes applied in chiral recognition.

The first example of the chiral recognition of inherently chiral calixarene was reported by Jin [63]. The fluorescence properties of two enantiomeric antipodes of tri-*O*-alkylated fluorescent chiral calix[4]arene **57** (Figure 22) with two pyrene moieties forming an intramolecular pyrene excimer towards three chiral guests were examined in CHCl_3_–EtOH (30:1) at 25 °C. The intensity of the excimer emission of **57** (3.9 μmol·dm^−3^) increased up to two-fold with the addition of *L*-phenylalanine methyl ester, l-alanine methyl ester, or l-phenylglycinol in the presence of Na^+^ (76 μmol·dm^−3^). However, differences in the fluorescence intensities between the two isomers of **57** in the presence of the chiral guests were too small for evaluating their chiral discriminating ability.

A series of tri-*O*-alkylated inherently chiral fluorescent calix[4]crowns in cone conformations and a series of tetra-*O*-alkylated inherently chiral fluorescent calix[4]crowns in partial cone conformations have been synthesized by Huang and Chen. Their recognition properties towards chiral aminoalcohols were measured in fluorescence titration experiments. The results indicated that two enantiomeric antipodes of **58** (Figure 22) have enantioselective recognition capability towards chiral leucinol. The association constant *Ka* of (+)-**58** in 1:1 stoichiometry was estimated to be 50 M^−1^ for d-leucinol and 143 M^−1^ for l-leucinol according to the Stern-Volmer plot [64].

A pair of enantiomeric antipodes of inherently chiral calix[4]arene **29** (Figure 12) containing amino phenol structures was synthesized by Shimizu. ^1^H NMR studies of the chiral calix[4]arene (+)-**29** with equimolar amounts of racemic manderic acid were performed. As a result of diastereomeric complexation, clear signal splitting with upfield shift of the benzilic proton of racemic mandelic acid was observed. Different proportions of both enantiomers of mandelic acid were also treated with (+)-**29**, and different signal intensities for both (*R*)- and (*S*)-mandelic acid, depending on the proportions, were observed. These results clearly indicated that inherently chiral calix[4]arene **29** could be used as a chiral NMR solvating agent for determining the enantiopurity of mandelic acid at ambient temperature. Furthermore, the recognition capability of (+)-**29** towards (*R*)- or (*S*)-mandelic acid was measured in a UV/Vis titration experiment. The association constant *Ka* of (+)-**29** with (*S*)- mandelic acid (3.5 × 105 dm^−3^mol^−1^) was 2.2-fold larger than that of (*R*)-mandelic acid (1.6 × 105 dm^−3^mol^−1^) [40,65].

Two enantiomers of inherently chiral calixarene carboxylic acid **59** (Figure 22) were synthesized by Kalchenko. The chiral recognition properties of (−)-**59** towards l and d α-phenylethylamine have been investigated using the ^1^H NMR technique. The results indicated the formation of the diastereomeric ammonium salt between (−)-**59** and l or d α-phenylethylamine, and their spectra have many differences. Consequently, calixarene carboxylic acid (−)-**59** can discriminate between the l and d enantiomers of α-phenylethylamine in the NMR spectrum [66].

### 4.2. Asymmetric Catalysis of Inherently Chiral Calixarenes

**Figure 23:**
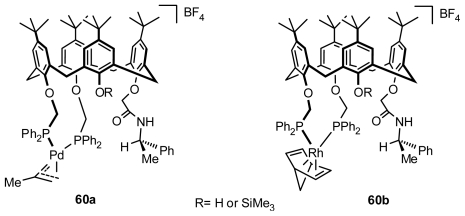
Palladium-(2-Me-allyl) and rhodium-(norbornadiene) complexes of inherently chiral calixarene bearing two phosphorus pendent groups.

Two pairs of enantiomeric palladium-(2-Me-allyl) and rhodium-(norbornadiene) complexes of inherently chiral calixarenes bearing two phosphorus pendent groups, **60a** and **60b** (Figure 23), were synthesized by Matt and display good activities when used as allylic alkylation and hydrogenation catalysts, respectively. Enantioselectivity was shown to depend on the size difference between another two auxiliary substituents. The catalytic properties of the ligands have been compared with those of related diphosphines, in which the only sources of chirality are asymmetric carbon atoms belonging to the auxiliary groups. The results clearly indicated that with regard to activity, the calixarenes that possess two proximal -CH_2_PPh_2_ groups are superior to those having the phosphines appended to distal positions. Moreover, the inherently chiral calixarenes gave enantioselectivities significantly higher than those induced by the other ligands studied, suggesting that a chiral calix skeleton is able to effectively transfer the chiral information to the catalytic center, whereas the presence alone of chiral C atoms located within the side chains has no significant influence on selectivity. Increasing the size difference between another two auxiliary substituents should likely result in higher ee’s [67]. This is the first example of asymmetric catalysis based on metal complexes of inherently chiral calix[4]arenes, which prompted us to look for more applications, although the results have been unsatisfactory. Recently, several transition-metal complexes of inherently chiral calix[4]arene-derived salphen ligands were also synthesized by Huang and Chen [68], but until now there has been no report of their application.

**Figure 24:**
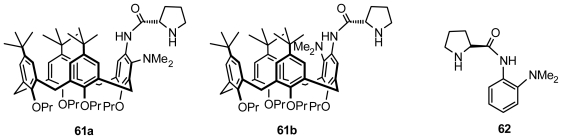
*m*-Dimethylamino-substituted inherently chiral calix[4]arenes bearing an l-prolinamido group and reference compound.

**Scheme 1:**
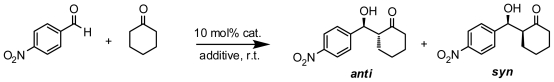
Asymmetric aldol reaction of 4-nitrobenzaldehyde and cyclohexanone.

Huang and Chen synthesized a pair of diastereomeric pure *m*-dimethylamino-substituted inherently chiral calix[4]arenes bearing an l-prolinamido group, **61a** and **61b** (Figure 24), via two routes and found that both could be utilized as bifunctional organocatalysts to efficiently promote the aldol reactions between aromatic aldehydes and ketones, such as 4-nitrobenzaldehyde and cyclohexanone, in the presence of acetic acid (Scheme 1). Especially with **61a** as the catalyst, the reaction between 4-nitrobenzaldehyde and cyclopentanone at −20 °C yielded an *anti*-aldol product up to 94% ee, whereas the *anti*-aldol product up to 94:6 dr and 79% ee was obtained when 4-cyanobenzaldehyde was used as the aldol donor. Moreover, in studies of the effect of temperature on the aldol reaction and comparison with the l-prolinamide derivative **62** (Figure 24) as the catalyst, they also found that the inherently chiral calixarene skeleton with a (*cS*)-conformation in **61a** was identified as the matched configuration of the stereogenic elements. The inherently chiral moiety might play an important role in stereocontrolling the reaction [69].

**Figure 25:**
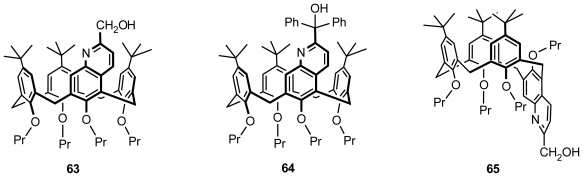
*N*,*O*-type chiral ligands derived from enantiopure inherently chiral calix[4]arenes containing a quinolin-2-yl-methanol moiety in cone or partial cone conformation.

A series of novel *N*,*O*-type chiral ligands, **63**, **64**, and **65** (Figure 25), derived from enantiopure inherently chiral calix[4]arenes containing a quinolin-2-yl-methanol moiety in cone or partial cone conformation, were synthesized and applied to asymmetric catalysis by Huang and Chen. The catalytic results indicated that these chiral ligands could catalyze the asymmetric addition of diethylzinc to benzaldehyde with high catalytic activity, although the enantioselectivity was low [70].

**Figure 26:**
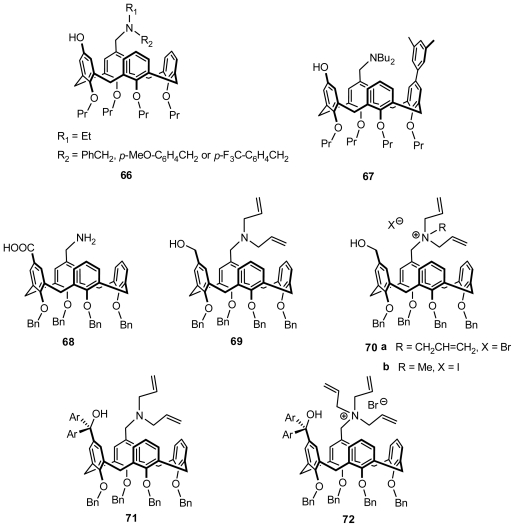
Inherently chiral calix[4]arenes containing amino acid structure, inherently chiral calix[4]arenes containing amino alcohol structure, and their quaternary ammonium salt.

**Scheme 2:**
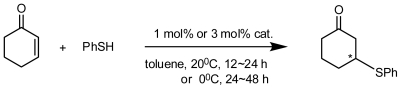
Asymmetric Michael addition reaction of thiophenol catalyzed by inherently chiral calix[4]arenes containing amino alcohol structure.

In addition to a pair of enantiomeric antipodes of inherently chiral calix[4]arene **29** (Figure 12) containing amino phenol structures, several of their analogs in **66** (Figure 26) were synthesized and optically resolved by the Shimizu group. In a preliminary asymmetric catalysis trial, an asymmetric Michael addition reaction of thiophenol (Scheme 2) was selected as a model reaction. Both enantiomers of **29** promoted the reaction efficiently and yielded a Michael addition product in excellent yields with a low ee, whereas some chiral induction was observed. The reaction at a lower temperature led to a slight increase in enantioselectivity, with no loss of reactivity. Subsequently, these analogs **66** were also examined as catalysts in the reaction but had poor selectivity regardless of the electronic properties of the benzyl substituent in the tertiary amine on the catalyst. Other substrates were also subjected to the Michael-type addition reactions, and similar results were observed [40,65].

To improve enantioselectivity of inherently chiral calix[4]arene catalysts, Shumizu introduced a 3,5-dimethylphenyl group on the wide rim as a sterically bulky substituent to prepare inherently chiral calix[4]arene **67** (Figure 26). The effect of the 3,5-dimethylphenyl group at the wide rim of calix[4]arene **67** on enantioselectivity was examined in asymmetric Michael addition reactions of thiophenol (Scheme 2), and a positive effect of the 3,5-dimethylphenyl group was observed. The author suggested that this result indicates a method of designing more efficient inherently chiral calixarene catalysts, although the asymmetric induction observed for the Michael addition reaction was still moderate [71].

**Scheme 3:**
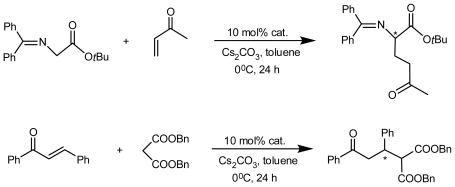
Asymmetric Michael addition reaction catalyzed by quaternary ammonium salts of inherently chiral calix[4]arenes containing amino alcohol structure under phase-transfer conditions.

A pair of enantiomers of inherently chiral calix[4]arene amino acid **68** (Figure 26), asymmetrically substituted on the upper rim, were synthesized by the Shimizu group and used as catalyst in an asymmetric direct aldol reaction of acetone. Unfortunately, no catalytic activity of chiral calix[4]arene **68** was observed, presumably because of the low nucleophilicity of the nitrogen on the calix[4]arene. Subsequently, a novel inherently chiral calix[4]arene containing an amino alcohol structure **69** and its quaternary ammonium salts **70a** and **70b** (Figure 26) were transformed from the separated optically pure derivatives of **68** with an *R*-BINOL moiety. Similar to those inherently chiral calix[4]arene amino alcohols reported above, **69** was also applied to an asymmetric Michael addition reaction of thiophenol (Scheme 2). Both enantiomers (+)-**69** and (−)-**69** promoted the reaction efficiently and provided a Michael addition product in good yield. The chiral induction of the product was observed as 15% ee, and the configuration of the major enantiomer was *S* with (+)-**69** and *R* with (−)-**69**. The quaternary ammonium salt of inherently chiral calix[4]arene was first applied to an asymmetric Michael addition reaction as asymmetric phase-transfer catalysis (Scheme 3). In a preliminary trial, they were applied to an asymmetric Michael addition reaction of a glycine derivative. Thus, the asymmetric Michael addition of a glycine derivative with methyl vinyl ketone in toluene and solid Cs_2_CO_3_ under the influence of either (+)-**70a** or (−)-**70a** gave the corresponding α-amino acid derivative in excellent yields with low enantioselectivities (5% ee). The reaction under the influence of **70b** provided a targeted product in excellent yield with slightly low selectivity compared with the reaction using **70a**. Asymmetric Michael addition reactions of malonate catalyzed by **70a** and **70b** were also examined under similar reaction conditions. In the case of Michel additions of malonate, catalyst **70b** was better than **70a** in terms of enantioselectivity. These results may indicate that the tuning of the catalyst structure in each reaction is important for the improvement of enantioselectivity [72].

The improvement of the design of inherently chiral calix[4]arenes as organocatalysts was accomplished via the introduction of a diarylmethanol structure [73]. Novel inherently chiral calix[4]arenes, **71** and **72** (Figure 26), bearing a tertiary amine or a quaternary ammonium moiety together with a diarylmethanol moiety, were synthesized in an optically pure form. They were then applied to asymmetric reactions as organocatalysts, and a positive effect of the diarylmethanol structure on enantioselectivity was observed. The asymmetric induction observed for the reaction remained moderate; however, this result might indicate the direction that the design of more efficient, inherently chiral calixarene catalysts should take.

## 4. Conclusions

In conclusion, we exhaustively discussed and classified the synthesis and optical resolution of many varieties of inherently chiral calixarenes and enumerated their application in chiral recognition and asymmetric catalysis. Tempting prospects in the study of inherently chiral calixarenes have been spawned from achievements in their synthesis, optical resolution, and applications. However, most of the exciting results are seriously limited in inherently chiral calix[4]arenes. Therefore, more in-depth studies of larger inherently chiral calixarenes are necessary in the future.
